# Unplanned Admission Following Day Surgery: A Retrospective Analysis of Rates, Causes, and Associated Risk Factors at a Single Center

**DOI:** 10.7759/cureus.83830

**Published:** 2025-05-10

**Authors:** Bandar M Almutairi, Sumayah A Althunayan, Nibras M Alamro, Jenan K Alqurishi, Shadan H Alfuraih, Shada S Aljumah, Abdulelah S Alharbi, Omar N Alharbi

**Affiliations:** 1 General Surgery, Buraidah Central Hospital, Buraidah, SAU; 2 College of Medicine, Qassim University, Buraidah, SAU

**Keywords:** conversion rate, day surgery, inpatient admission, retrospective study, surgical procedures

## Abstract

Background

Day surgery, also known as same-day surgery, involves admitting and discharging patients on the same day, aiming to reduce hospital stays. However, various factors can necessitate unplanned inpatient admission.

This study assessed admission rates following day surgery and evaluated the causes and associated risk factors, including patient characteristics, type of anesthesia, type of surgery, and time of arrival at the day surgery unit, at Buraidah Central Hospital, Qassim, Saudi Arabia.

Methods

A retrospective cohort study was conducted in Buraidah Central Hospital, Qassim, Saudi Arabia, using medical records of patients who underwent day surgery between January 2022 and December 2023. Data on demographics, cause of admission, comorbidities, surgery type, anesthesia, and arrival time at the day surgery unit were analyzed.

Results

Of 3,806 patients who underwent day surgery during the study period, 293 (7.6%) were converted to inpatient care. Most admitted patients were aged 18-59 years, men, and overweight. The primary reasons for admission were the need for further observation and close monitoring. Laparoscopic cholecystectomy was the most common procedure, followed by adenotonsillectomy. Women undergoing laparoscopic cholecystectomy had a higher conversion rate than men. Adenotonsillectomy was more common among patients admitted due to complications.

Conclusion

The conversion rate from day surgery to inpatient care was 7.6%, higher than the rates reported in other hospitals. This may be linked to the high prevalence of laparoscopic cholecystectomy, which had an 18.3% local conversion rate. General anesthesia was associated with higher conversion rates. No intensive care unit (ICU) admissions were recorded, suggesting severe complications were rare despite the conversions.

## Introduction

Day surgery, or same-day surgery, involves admitting and discharging patients on the same day and has gained popularity due to efficiency, patient demand, and advances in surgical and anesthetic techniques [1,2]. It offers benefits like reduced costs, quicker recovery, shorter waiting lists, and lower infection risk, but early discharge can lead to issues like pain, nausea, and dizziness [3-6].

This approach is common in specialties like ophthalmology, orthopedics, and ear, nose, and throat (ENT), while less frequent in general and vascular surgery [7,8]. In Buraidah, Saudi Arabia, common procedures include pilonidal sinus excision, hernia repair, and laparoscopic cholecystectomy [3].

Careful patient selection is crucial, considering surgical, medical, and social factors [9]. Stable patients with well-managed chronic conditions are ideal candidates, while obesity and obstructive sleep apnea (OSA) require special precautions [9,10]. Social considerations include obtaining informed consent and ensuring a responsible adult stays with the patient post-surgery [11-13].

Low-risk procedures with minimal complications are preferred for day surgery [9]. Common reasons for inpatient conversion include pain, bleeding, and postoperative care needs, as seen in studies from Belfast (1991) and King Fahad Specialist Hospital in Buraidah (2021) [2,3].

This study aims to assess the conversion rate from day surgery to inpatient admission at Buraidah Central Hospital and evaluate associated risk factors, including patient demographics, type of surgery, type of anesthesia, and arrival time at the day surgery unit.

## Materials and methods

This retrospective cohort study was conducted at Buraidah Central Hospital in Qassim, Saudi Arabia. Ethical approval was obtained from the Qassim Regional Research Ethics Committee and the hospital administration (approval number: 607-46-4072)

All patient data were anonymized and de-identified prior to analysis to ensure confidentiality. The study involved no direct interaction with the patients, and the data were used solely for research purposes.

This study included all patients who underwent day surgery between January 1, 2022, and December 31, 2023 (n = 3,806). Patients were excluded if they were admitted to the hospital for their surgery and underwent day surgery prior to January 2022 or after December 2023.

Data were extracted using a standardized Excel (Microsoft Corp., Redmond, WA, USA) sheet, which reviewed medical records, including operative reports, admission details, and post-discharge records. The following variables were collected: conversion to inpatient admission, primary reasons for conversion, age, gender, body mass index (BMI), presence of comorbidities, type of surgery performed, type of anesthesia used, and time of patient arrival at the day surgery unit.

All data extracted followed uniform hospital documentation standards, ensuring comparability across groups. 

The collected data were analyzed using IBM SPSS Statistics for Windows, Version 26.0 (Released 2019; IBM Corp., Armonk, NY, USA). Categorical variables were expressed as frequencies and percentages. The chi-squared test was used to evaluate the relationship between variables. A p < 0.05 was considered statistically significant. Missing data were mentioned in each category as NA.

## Results

**Figure 1:**
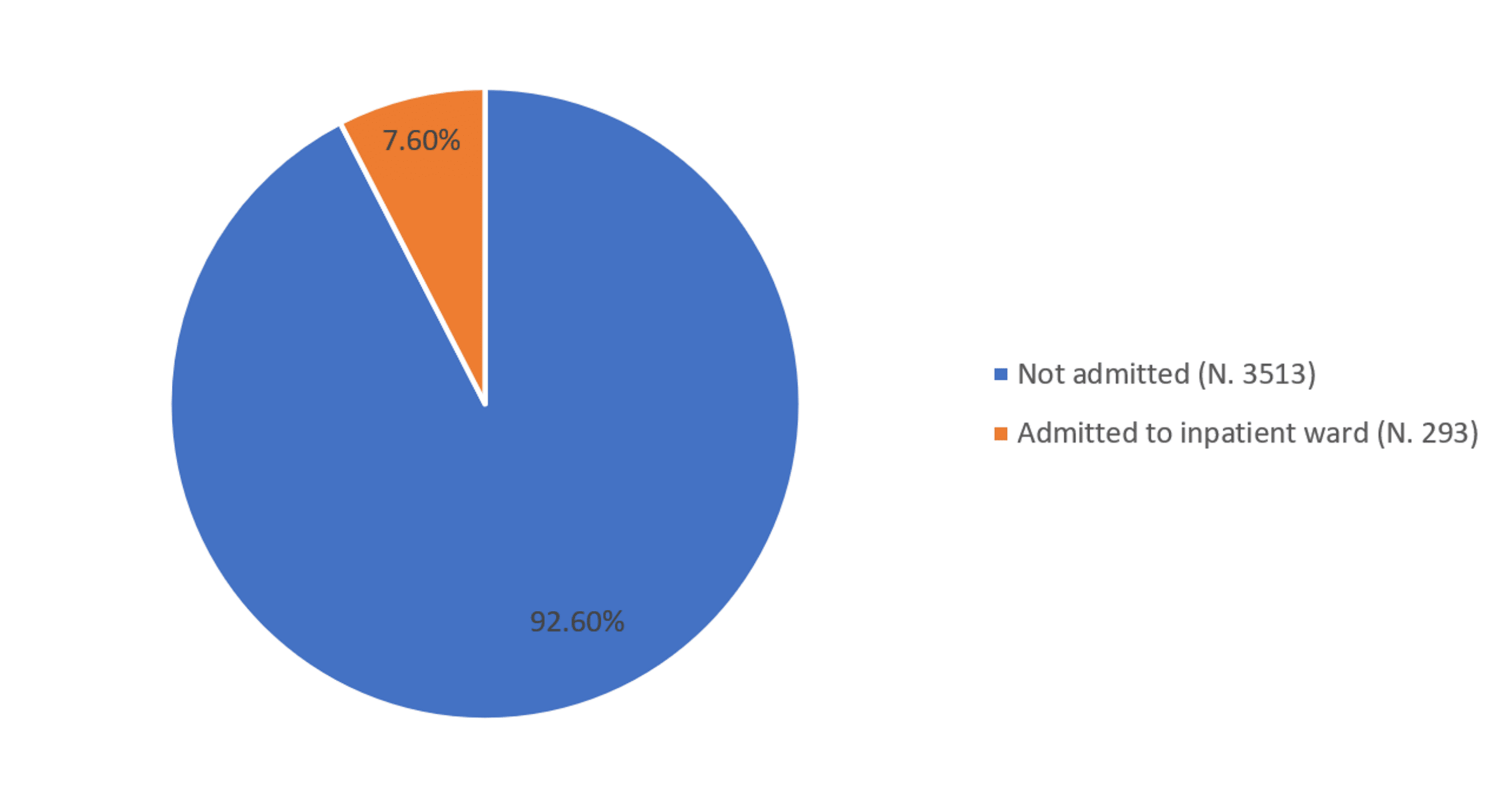
Rate of patient conversion to inpatient wards at our hospital (January 1, 2022-December 31, 2023)

Of the 3,806 patients who underwent day surgery at our hospital between January 1, 2022, and December 31, 2023, 293 (7.6%) were admitted to inpatient wards (Figure 1).

Among the 293 patients who were admitted, 66.6% were aged 18-59 years, 54.3% were men, and 40.3% were classified as overweight (Table 1).

**Table 1:** Distribution of patients according to their demographic characteristics and BMI (N = 293)

Variable	N (%)
Age	
<18 years old	60 (20.5)
18-59 years old	195 (66.6)
>60 years old	22 (7.5)
NA	16 (5.5)
Gender	
Male	159 (54.3)
Female	119 (40.6)
NA	15 (5.1)
BMI	
Underweight	8 (2.7)
Normal	67 (22.9)
Overweight	118 (40.3)
Obese	23 (7.8)
Healthy weight (child)	30 (10.2)
Overweight (child)	22 (7.5)
NA	25 (8.5)

**Figure 2:**
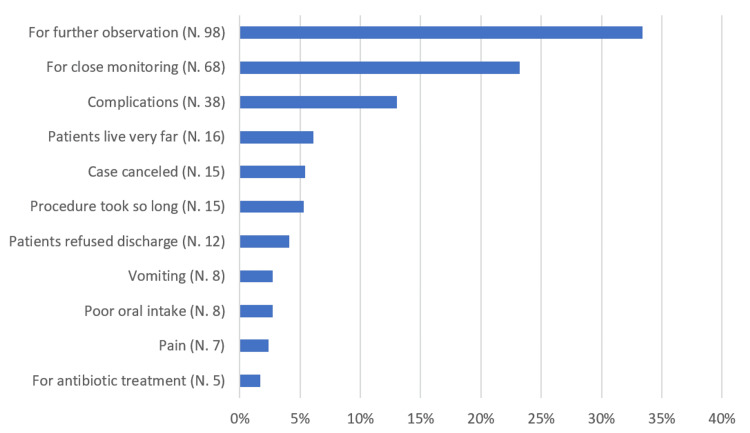
Percentage distribution of causes for conversion to inpatient admission (N = 293)

**Figure 3:**
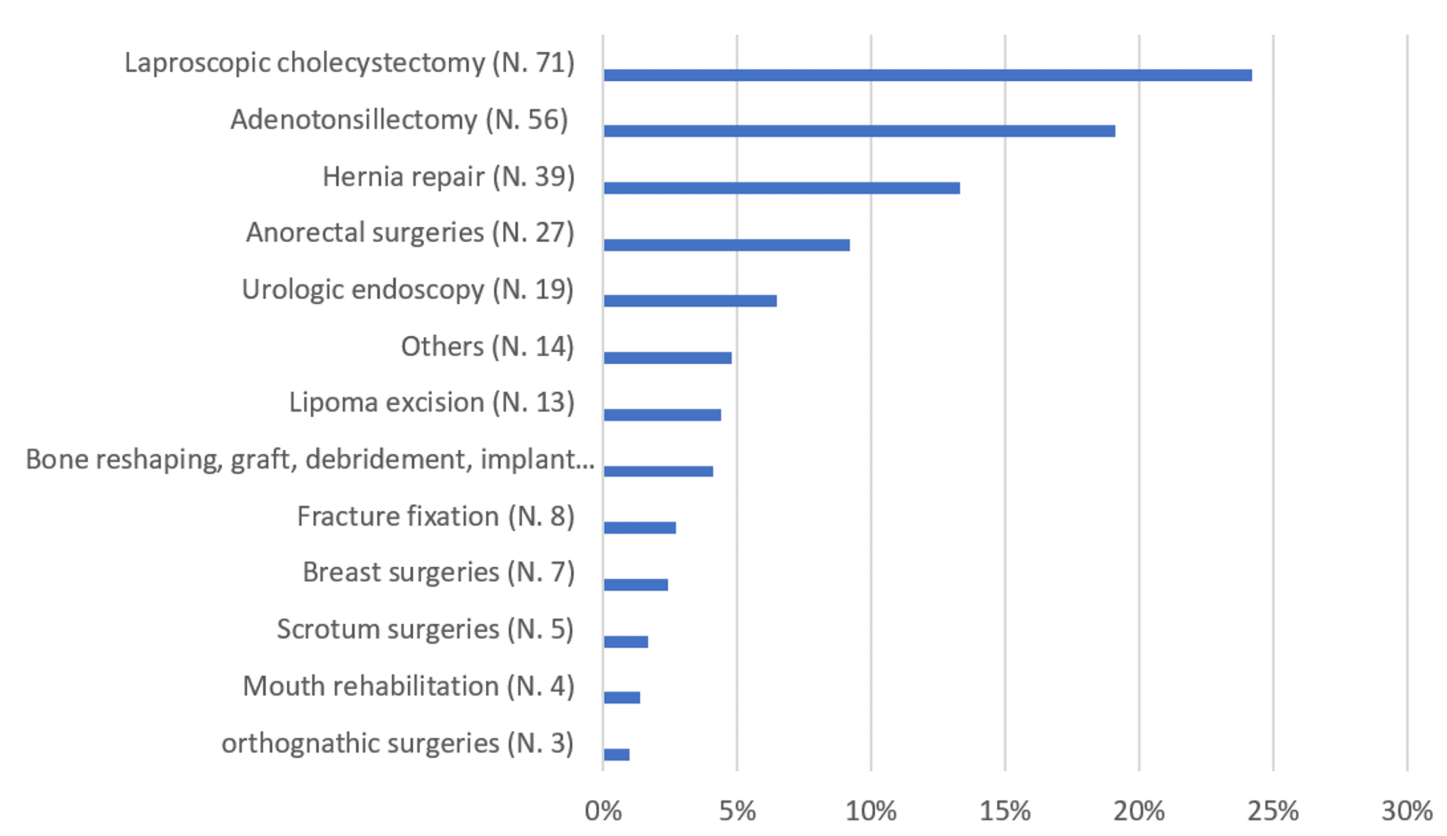
Percentage distribution of patients according to the type of surgery (N = 278)

As shown in Table 2, Figure 2, and Figure 3, the most common cause of conversion to inpatient admission was the need for further observation (33.4%), followed by the need for close monitoring (23.2%). Among the participants, 9.2% had comorbidities, with hypertension (HTN) (4.1%) and diabetes mellitus (DM) (3.8%) being the most prevalent. Laparoscopic cholecystectomy (24.2%) was the most commonly performed surgery, followed by adenotonsillectomy (19.1%) and hernia repair (13.3%). Spinal anesthesia was used in 7.8% of the documented cases. Regarding the time of arrival at the day surgery unit, 27% of patients were received from the operating room (OR) between 9:00 AM and 10:59 AM, 23.9% between 11:00 AM and 12:59 PM, and 20.8% between 1:00 PM and 2:59 PM.

**Table 2:** Distribution of patients according to causes of conversion to inpatient admission, clinical data, and time of arrival at the day surgery unit from the OR (N = 293) DM: diabetes mellitus, HTN: hypertension, GA: general anesthesia, OR: operating room.

Variable	N (%)
Cause of conversion	
For further observation	98 (33.4)
Case canceled	16 (5.4)
Vomiting	8 (2.7)
Patient lives very far	18 (6.1)
Patient refused discharge	12 (4.1)
For close monitoring	68 (23.2)
For antibiotic treatment	5 (1.7)
Complications	38 (13)
Pain	7 (2.4)
Poor oral intake	8 (2.7)
Procedure took so long	15 (5.3)
Comorbidity	
DM	11 (3.8)
HTN	12 (4.1)
Hypothyroidism	4 (1.4)
Cancer	1 (0.3)
Renal insufficiency	1 (0.3)
Asthma	6 (2)
No comorbidity	258 (88)
Type of surgery	
Lipoma excision	13 (4.4)
Laparoscopic cholecystectomy	71 (24.2)
Hernia repair	39 (13.3)
Breast surgeries	7 (2.4)
Adenotonsillectomy	56 (19.1)
Scrotum surgeries	5 (1.7)
Urologic endoscopy	19 (6.5)
Others	14 (4.8)
Fracture fixation	8 (2.7)
Bone reshaping, graft, debridement, implant removal	12 (4.1)
Mouth rehabilitation	4 (1.4)
Orthognathic surgeries	3 (1)
Anorectal surgeries	27 (9.2)
NA	15 (5.1)
Type of anesthesia	
GA	251 (85.7)
Local	2 (0.7)
Spinal	23 (7.8)
NA	17 (5.8)
Time of arrival at the day surgery unit from the OR	
9:00 AM and 10:59 AM	79 (27)
11:00 AM and 12:59 PM	70 (23.9)
1:00 PM and 2:59 PM	61 (20.8)
300 PM and 5:00 PM	58 (19.8)
NA	25 (8.5)

Table 3 shows that women had a significantly higher percentage of undergoing laparoscopic cholecystectomy compared to men (58.8% vs. 27.9%; p < 0.05). On the other hand, no statistically significant gender differences were observed for the three most common causes of conversion (p > 0.05).

**Table 3:** Gender distribution of patients according to the most common causes of conversion to inpatient admission and type of surgery (N = 293) NB: χ^2^ = 18.3, p < 0.001.

Variable	Gender		χ^2^	p-value
	Male (N, %)	Female (N, %)		
Top three causes of conversion				
For further observation	61 (51.7)	37 (43)	3.63	0.162
For close monitoring	33 (28)	35 (40.7)		
Complications	24 (20.3)	14 (16.3)		
Top three types of surgery				
Laparoscopic cholecystectomy	24 (27.9)	47 (58.8)	18.3	<0.001
Hernia repair	29 (33.7)	10 (12.5)		
Adenotonsillectomy	33 (38.4)	23 (28.7)		

**Figure 4:**
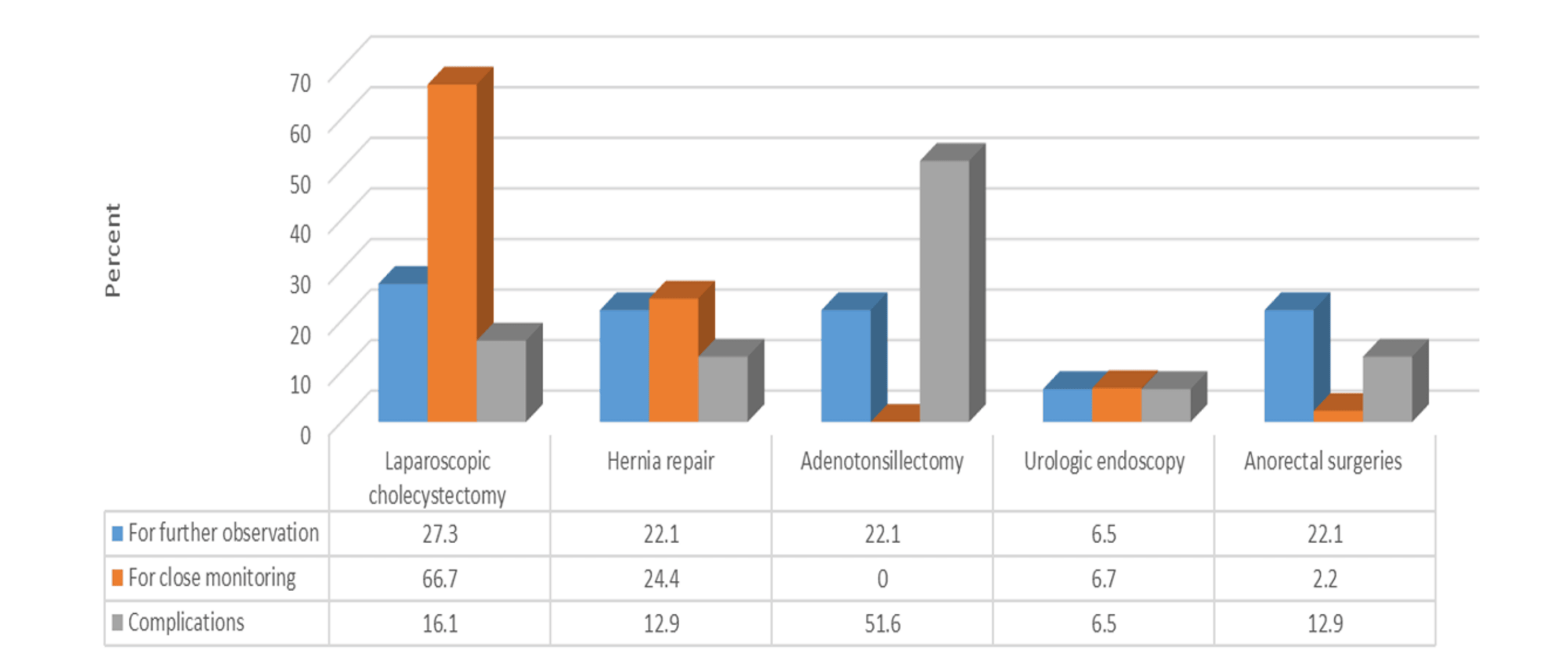
Graphical representation of the relationship between the three most common causes of conversion to inpatient admission and the five most common types of surgery NB: χ^2^ = 48.3, p < 0.001.

Table 4 and Figure 4 demonstrate that patients admitted due to complications had a significantly higher percentage of undergoing adenotonsillectomy surgery. In contrast, patients admitted for close monitoring had a significantly higher percentage of undergoing laparoscopic cholecystectomy (p < 0.05).

**Table 4:** Relationship between the three most common causes of conversion to inpatient admission and the five most common types of surgery (N = 293)

Variable	Top three causes of conversion			χ^2^	p-value
	For further observation (N, %)	For close monitoring (N, %)	Complications N. (%)		
Top five types of surgery					
Laparoscopic cholecystectomy	21 (27.3)	30 (66.7)	5 (16.1)	48.3	<0.001
Hernia repair	17 (22.1)	11 (24.4)	4 (12.9)		
Adenotonsillectomy	17 (22.1)	0 (0.0)	16 (51.6)		
Urologic endoscopy	5 (6.5)	3 (6.7)	2 (6.5)		
Anorectal surgeries	17 (22.1)	1 (2.2)	4 (12.9)		

## Discussion

This study assessed the conversion rate of day surgeries to inpatient admissions, with a reported rate of 7.6%. Several factors contributed to unplanned admissions in our study, reflecting both clinical needs and logistical considerations. Some patients require extended observation due to underlying chronic conditions, the need for multidisciplinary consultations, or ongoing wound care. In other cases, persistent postoperative symptoms such as vomiting or severe pain prevented safe discharge. Geographic factors also played a role; patients living far from the hospital, particularly in remote areas of Qassim, were often admitted, minimizing the risks associated with delayed access to emergency care.

Operational challenges, such as overbooked surgical lists, led to case cancellations and rescheduling, which occasionally resulted in inpatient stays. Additionally, some patients declined discharge due to feelings of weakness or fear of complications, and their concerns were accommodated with short-term admissions. Clinical indicators such as abnormal vital signs, poor oral intake, or the need to monitor surgical drains and nasal packing also necessitated closer inpatient monitoring.

Complications during or after surgery, including bleeding, infections, hypoxia, and convulsions, were among the more serious causes of admission. Prolonged procedures and intraoperative difficulties, often related to technical or equipment issues, further extended the recovery time. The diversity and complexity of procedures performed, such as laparoscopic cholecystectomies, orthognathic surgeries, and fracture fixations, added to the overall admission risk. While day surgeries were typically scheduled earlier to allow for postoperative evaluation, certain cases still required inpatient care despite these precautions.

The reported rate of 7.6%, which was used to evaluate the conversion rate of day surgeries to inpatient hospitalizations, is much higher than prior research. For instance, studies conducted in Singapore and Belgium reported rates of 1.5% [5] and 2.89% [14], respectively. At the Duke University Medical Center, the conversion rate was as low as 0.11% [15]. The relatively high conversion rate in our study can be attributed to the significant proportion of laparoscopic cholecystectomies, which constituted 24.2% of the sample. Locally, this procedure is associated with a high conversion rate of 18.3% [3,16]. Other studies similarly found conversion rates ranging between 9.8% and 11.2% for laparoscopic cholecystectomy patients [6,17].

The leading cause of admission in our study was further observation (33.4%), followed by close monitoring (23.2%) and surgical complications (13%). In contrast, other studies found surgically related causes, such as bleeding and pain, to account for the majority of unplanned admissions (58.3%-62.8%) [5,17]. In the USA, unplanned admissions were primarily attributed to surgical causes (55%), medical causes (22%), and anesthesia-related causes (8%) [18]. Locally, a study conducted at King Fahad Specialist Hospital also reported surgical complications as a primary reason for admission (43.8%) [3].

The type of anesthesia used was another important factor in unplanned admissions. Patients receiving general anesthesia had a higher conversion rate than those who received local or spinal anesthesia. This finding aligns with a previous research, such as a study on outpatient arthroscopic shoulder surgeries, which reported higher admission rates with general anesthesia [19]. This may be explained by the more invasive nature of surgeries requiring general anesthesia [15].

While our study reported no ICU admissions, severe complications requiring ICU care have been noted in other studies. Early detection and management of complications using tools like point-of-care ultrasound (POCUS) could prevent delays and improve outcomes during the transfer process [20-23].

Limitations

This study has several limitations that should be acknowledged. Missing records and incomplete patient files limited the accuracy and comprehensiveness of the dataset. As a study conducted in a single center in Saudi Arabia, the findings may not be generalizable to a broader or more diverse populations. Not all-day surgery cases during the study period were accessible, potentially introducing selection bias. Addressing these limitations in future research could provide a more robust understanding of the rates, causes, and associated factors of day surgery conversions.

## Conclusions

This retrospective study identified a 7.6% conversion rate from day surgery to inpatient admission, which is higher than rates reported in other hospitals. This elevated rate is likely due to the high proportion of laparoscopic cholecystectomy cases (24.2%) in the sample, with a local conversion rate of 18.3%. Additionally, the use of general anesthesia was associated with higher conversion rates compared to local or spinal anesthesia. Importantly, no ICU admissions were recorded, suggesting that severe postoperative complications were rare despite the higher conversion rate.

Future studies should explore strategies to reduce conversion rates, such as refining patient selection criteria and enhancing postoperative monitoring protocols, particularly for high-risk procedures like laparoscopic cholecystectomy.
